# Overall treatment time and oncologic outcomes after definitive radiotherapy or chemoradiotherapy for cervical cancer: a systematic review and meta-analysis

**DOI:** 10.3389/fonc.2026.1969525

**Published:** 2026-10-26

**Authors:** Yingding Ruan, Yun Li, Dan Wu, Jianwei Han, Jianxia Cheng

**Affiliations:** 1 Department of Thoracic Surgery, The First People’s Hospital of Jiande, Jiande, China; 2 Department of Gynecology, The First People’s Hospital of Jiande, Jiande, China

**Keywords:** chemoradiotherapy, duration of therapy, radiotherapy, treatment outcome, uterine cervical neoplasms

## Abstract

**Background:**

Prolonged overall treatment time (OTT) during definitive radiotherapy has long been considered detrimental in cervical cancer, but the magnitude and consistency of its prognostic association across treatment eras remain uncertain. We evaluated the association between OTT and oncologic outcomes after definitive radiotherapy or chemoradiotherapy.

**Methods:**

PubMed/MEDLINE, Embase, Web of Science Core Collection, Scopus, and the Cochrane Library were searched from inception through August 1, 2026. Eligible studies assessed OTT from initiation of external-beam radiotherapy through completion of the planned definitive radiotherapy course as either a categorical or continuous prognostic factor. Risk of bias was assessed using the Quality In Prognosis Studies tool. Time-to-event estimates were pooled as hazard ratios (HRs) with 95% confidence intervals (CIs) using random-effects models.

**Results:**

Nineteen studies comprising 15,859 patients were included. Ten independent cohorts contributed to the primary overall survival (OS) analysis. Prolonged OTT was associated with poorer OS (HR, 1.61; 95% CI, 1.30–1.99; I² = 76%). In the prespecified analysis of three independent cohorts comparing OTT >56 versus ≤56 days, the association persisted (HR, 1.16; 95% CI, 1.04–1.31; I² = 37%). Prolonged OTT was also associated with a higher risk of local/pelvic/in-field failure (HR, 1.80; 95% CI, 1.26–2.56; I² = 46%). The OS association was consistent across treatment-era and cutoff-source subgroups and remained robust in sensitivity analyses.

**Conclusion:**

Prolonged OTT is associated with poorer survival and a higher risk of local/pelvic/in-field failure after definitive radiotherapy or chemoradiotherapy for cervical cancer. These findings support timely completion of definitive radiotherapy and indicate that the 56-day interval remains a clinically relevant benchmark.

**Systematic review registration:**

https://www.crd.york.ac.uk/PROSPERO/view/CRD420261470588, identifier CRD420261470588.

## Introduction

Cervical cancer remains a major cause of cancer-related morbidity and mortality among women worldwide. The latest GLOBOCAN 2024 estimates indicate approximately 604,000 new cases and 280,000 deaths globally, with a disproportionate burden in countries with limited access to prevention and high-quality cancer treatment (1). Definitive radiotherapy is delivered with curative intent as the primary local treatment rather than following primary surgery and generally comprises external-beam radiotherapy (EBRT), concurrent platinum-based chemotherapy, and brachytherapy. It is recommended for patients with FIGO stage IB3–IVA disease and may also be used for medically inoperable patients with stage IA1–IB2 disease (2, 3). Magnetic resonance imaging–guided adaptive brachytherapy has further improved local disease control while limiting treatment-related morbidity (4). Recent phase III trials, including KEYNOTE-A18 and INTERLACE, illustrate the continuing evolution of treatment paradigms alongside definitive chemoradiotherapy and brachytherapy (5, 6). Against this background, the duration of definitive radiotherapy remains clinically relevant.

Overall treatment time (OTT) captures the elapsed duration and temporal integrity of the definitive radiotherapy course rather than simply the prescribed radiation dose. Prolongation is biologically concerning because continued tumor-cell repopulation during a protracted course may reduce the effectiveness of radiotherapy; consistent with this rationale, the RetroEMBRACE cohort showed that local control was jointly associated with tumor dose, tumor volume, and OTT (7). The conventional 8-week (56-day) benchmark originated from earlier observational evidence linking prolonged treatment with poorer oncologic outcomes (8), whereas contemporary recommendations use slightly shorter treatment-completion targets: the 2023 ESGO/ESTRO/ESP guidelines recommend completing EBRT and image-guided brachytherapy within 7 weeks, and the ESGO/ESTRO radiation therapy quality indicators define OTT ≤50 days as a quality target (3, 9).

Whether the conventional 8-week benchmark retains the same prognostic meaning in contemporary practice, however, remains uncertain given substantial advances in concurrent chemoradiotherapy and image-guided brachytherapy. Hong et al. specifically revisited this question in the concurrent chemoradiotherapy era and questioned whether 8 weeks remained the most informative treatment-duration target (10). Subsequent contemporary studies have yielded mixed findings. Treatment duration has remained prognostically relevant in cohorts incorporating magnetic resonance imaging–based brachytherapy (11), whereas a large database analysis suggested that completion of brachytherapy itself may have greater survival importance than adherence to the 8-week target alone (12). More broadly, the available evidence spans markedly different radiotherapy eras and varies in concurrent chemotherapy and brachytherapy use, OTT definitions, treatment-duration cutoffs, confounder adjustment, and reported oncologic endpoints. These differences complicate estimation of both the overall magnitude of the prognostic association and the consistency with which a clinically recognizable threshold such as 56 days can be applied across treatment settings. Although this literature has recently been reviewed (8), no previous meta-analysis, to our knowledge, has quantitatively pooled time-to-event effect estimates for OTT during definitive radiotherapy in cervical cancer, an exposure defined by treatment-course duration rather than delay before treatment initiation.

We therefore conducted a systematic review and meta-analysis to evaluate the association between OTT and oncologic outcomes in patients with cervical cancer treated with definitive radiotherapy or chemoradiotherapy. The primary objective was to quantify the association between prolonged OTT and overall survival; secondary objectives included evaluation of local, pelvic, or in-field failure and other oncologic outcomes when sufficient data were available. We also examined the clinically established 56-day benchmark using a uniform >56 versus ≤56-day comparison and explored whether treatment era, treatment mode, and OTT cutoff definition accounted for variation in the reported prognostic association.

## Methods

### Protocol and reporting

This systematic review and meta-analysis was reported in accordance with the Preferred Reporting Items for Systematic Reviews and Meta-Analyses (PRISMA) 2020 statement (13) and conducted with reference to the methodological guidance in the Cochrane Handbook for Systematic Reviews of Interventions (14). The review protocol was prospectively registered in the International Prospective Register of Systematic Reviews (PROSPERO; registration No. CRD420261470588). No substantive changes were made to the registered protocol; an outcome-level certainty-of-evidence assessment was added during manuscript revision. The methods are described below in the sequence of literature searching, eligibility assessment and study selection, data extraction, risk-of-bias and certainty assessment, and statistical analysis.

### Search strategy

A systematic literature search was conducted in PubMed/MEDLINE, Embase, Web of Science Core Collection, Scopus, and the Cochrane Library from database inception through August 1, 2026. The search strategy combined terms representing four concepts: cervical cancer; radiotherapy, chemoradiotherapy, or brachytherapy; overall treatment time (OTT), treatment duration, treatment prolongation, treatment interruption, treatment breaks, or treatment gaps; and oncologic outcomes or prognosis. Controlled-vocabulary and free-text terms were adapted to the indexing system and syntax of each database.

Specific OTT cutoff values, disease stage, age, histologic subtype, effect-measure terms, and study-design filters were not required search concepts. No publication-type exclusion filter was applied during the electronic search. Reference lists of eligible studies and relevant reviews or guidelines were also screened, and forward citation tracking was performed to identify additional potentially eligible reports. Complete database-specific search strategies are provided in the Supplementary Methods.

### Eligibility criteria

Eligible studies enrolled adults with primary cervical cancer treated with curative-intent definitive radiotherapy or chemoradiotherapy. Treatment could consist of external-beam radiotherapy (EBRT) alone or EBRT combined with intracavitary or interstitial brachytherapy, with or without concurrent chemotherapy. Mixed-population studies were eligible only when data for the relevant cervical cancer population and definitive treatment setting could be identified separately.

The exposure of interest was OTT across the definitive radiotherapy course. Eligible studies were required to assess treatment duration from initiation of definitive EBRT through completion of the planned radiotherapy course, including brachytherapy when it formed part of definitive treatment. Both categorical comparisons of longer versus shorter OTT and analyses treating OTT as a continuous prognostic factor were eligible.

The primary outcome was overall survival (OS). Local, pelvic, or in-field failure was the principal locoregional outcome. Other outcomes of interest included cancer-specific or disease-specific survival (CSS/DSS), disease-free survival (DFS), progression-free survival (PFS), recurrence-free survival (RFS), distant failure, and fixed-time survival or recurrence outcomes.

Eligible designs included prospective or retrospective cohort studies, registry- or database-based cohort studies, and secondary prognostic analyses of clinical trial data. Only peer-reviewed, full-text original studies published in English were eligible. Studies were excluded if they evaluated only pretreatment waiting time, isolated EBRT duration, the interval between EBRT and brachytherapy, or treatment interruptions without an assessable overall definitive treatment duration. Studies of exclusively postoperative or preoperative radiotherapy, palliative treatment, re-irradiation, or metastatic disease were excluded unless eligible definitive-treatment data were separately available. Reviews, systematic reviews or meta-analyses, editorials, case reports, conference abstracts or proceedings without eligible full-text data, dissertations, and preclinical studies were also excluded.

### Study selection

After duplicate records were removed, two reviewers independently screened titles and abstracts for eligibility. Potentially eligible full-text reports were then assessed against the prespecified criteria. Disagreements were resolved by discussion, with consultation of a third reviewer when consensus could not be reached.

Potentially overlapping study populations were compared by data source or participating center, enrollment period, patient population, treatment setting, OTT definition, and reported outcome. When multiple reports represented the same or substantially overlapping cohort and addressed the same outcome, the report providing the most informative eligible effect estimate was retained, considering completeness of follow-up, appropriateness of the OTT definition, sample size, and extent of multivariable adjustment. Reports from overlapping sources could contribute to different analyses when they provided distinct, nonduplicated information. Each underlying cohort contributed no more than one independent effect estimate to any individual meta-analysis.

### Data extraction

Two reviewers independently extracted data using a standardized form. Study-level variables included first author, publication year, country or region, study design, participating center or database, enrollment period, sample size, and follow-up duration. Patient and disease characteristics included age, FIGO stage, histologic subtype, and other reported clinicopathologic prognostic factors.

Treatment-related variables included definitive radiotherapy or chemoradiotherapy, concurrent chemotherapy use, EBRT technique and dose, brachytherapy use and technique, and reported treatment interruptions or delays. For OTT, we recorded the reported start and end points, duration, categorical cutoff values, cutoff-definition method, and continuous exposure definitions. Outcome definitions, effect estimates, 95% confidence intervals (CIs), and covariates included in multivariable models were extracted separately for each oncologic endpoint.

For time-to-event outcomes, multivariable-adjusted hazard ratios (HRs) and corresponding 95% CIs were preferentially extracted when available. When several eligible multivariable estimates were reported, we selected the model with the most clinically appropriate adjustment for baseline prognostic factors. Risk of bias assessment.

Risk of bias was assessed independently by two reviewers using the Quality In Prognosis Studies (QUIPS) tool (15), because OTT was evaluated as a prognostic factor rather than as a therapeutic intervention. The six QUIPS domains were study participation, study attrition, prognostic factor measurement, outcome measurement, study confounding, and statistical analysis and reporting.

Each domain was judged as having low, moderate, or high risk of bias based on information reported in the original study. Overall judgments reflected the methodological importance of individual domains rather than a numerical summary score. Disagreements were resolved by discussion and, when necessary, adjudication by a third reviewer.

In addition, the certainty of evidence was assessed at the outcome level for each quantitative synthesis, considering study limitations, inconsistency, indirectness, imprecision, and potential publication bias or small-study effects. Overall certainty was categorized as high, moderate, low, or very low, with disagreements resolved by discussion between the reviewers.

### Statistical analysis

Meta-analyses were conducted using Review Manager (RevMan), version 5.4. Time-to-event estimates were transformed to the natural logarithm of the HR, and corresponding standard errors were derived from the reported 95% CIs. Pooled HRs and 95% CIs were calculated using the generic inverse-variance method.

Because substantial clinical and methodological variation was expected across treatment eras, radiotherapy techniques, treatment regimens, OTT definitions, and cutoff values, random-effects models were used for all quantitative syntheses. Statistical heterogeneity was assessed with Cochran’s Q test and the I² statistic. A P value <0.10 for Cochran’s Q test was considered indicative of statistical heterogeneity, and I² was used to quantify between-study inconsistency.

Quantitative synthesis was performed when at least three independent studies provided clinically compatible estimates for the same outcome and effect-measure category. Outcomes that did not meet this criterion, or for which definitions or adjustment strategies were insufficiently comparable, were summarized descriptively rather than pooled solely to increase the number of studies in a meta-analysis.

Cox regression–derived relative risk estimates were treated as equivalent to HRs when their derivation and interpretation were consistent with a proportional-hazards analysis. When a study reported several categorical OTT comparisons, we preferentially selected a comparison based on the 56-day treatment interval when available; otherwise, the clinically defined or principal study cutoff was used, followed by a data-derived cutoff when no more appropriate estimate was available. Effect directions were harmonized so that an HR >1 consistently indicated a higher risk of an adverse oncologic outcome with longer OTT. Estimates reported in the opposite direction were inverted together with their confidence limits. Adjusted and unadjusted estimates, categorical and continuous OTT effects, and different oncologic outcomes were retained separately. When mathematically appropriate and unambiguous, continuous OTT estimates were standardized to a 7-day increment for descriptive comparison.

The primary quantitative analysis evaluated the association between study-defined prolonged OTT and OS. A separate clinically focused analysis was restricted to studies using the exact comparison of OTT >56 days versus ≤56 days. For locoregional outcomes, estimates of local, pelvic, and in-field failure were combined when their definitions were sufficiently comparable to represent failure within the definitive treatment region.

Subgroup analyses of OS were conducted by treatment era and treatment mode, source of the OTT cutoff, and magnitude of the study-specific OTT cutoff. Treatment-era analyses were performed according to the predominant treatment context reported in each original cohort. Historical radiotherapy-dominant/radiotherapy-alone cohorts represented studies in which radiotherapy without concurrent chemotherapy was the main treatment approach, whereas chemotherapy-containing/concurrent chemoradiotherapy cohorts represented studies with routine concurrent chemotherapy use. Cutoff-source analyses distinguished clinical, guideline-based, or protocol-prespecified thresholds from distribution-based or data-derived thresholds. Study-specific cutoff values were also categorized as ≤56 days, 57–63 days, or ≥64 days. Subgroup differences were evaluated using tests for interaction.

Sensitivity analyses included an expanded 56-day threshold-family analysis incorporating closely related boundary definitions, exclusion of studies at high overall risk of bias from the relevant primary synthesis, and leave-one-out analyses with sequential omission of individual studies. All statistical tests were two-sided, and P < 0.05 was considered statistically significant. Small-study effects were assessed by visual inspection of funnel plots and Egger’s regression test when a meta-analysis included at least 10 independent studies.

## Results

### Study selection and characteristics

The systematic search of PubMed/MEDLINE, Embase, Web of Science Core Collection, Scopus, and the Cochrane Library identified 1,770 records. After removal of 699 duplicates, 1,071 records underwent title and abstract screening, of which 144 were excluded. The remaining 927 reports were assessed for full-text eligibility; 908 were excluded, leaving 19 studies (10, 11, 16–32) for inclusion in the systematic review (**Figure 1**).

**Figure 1:**
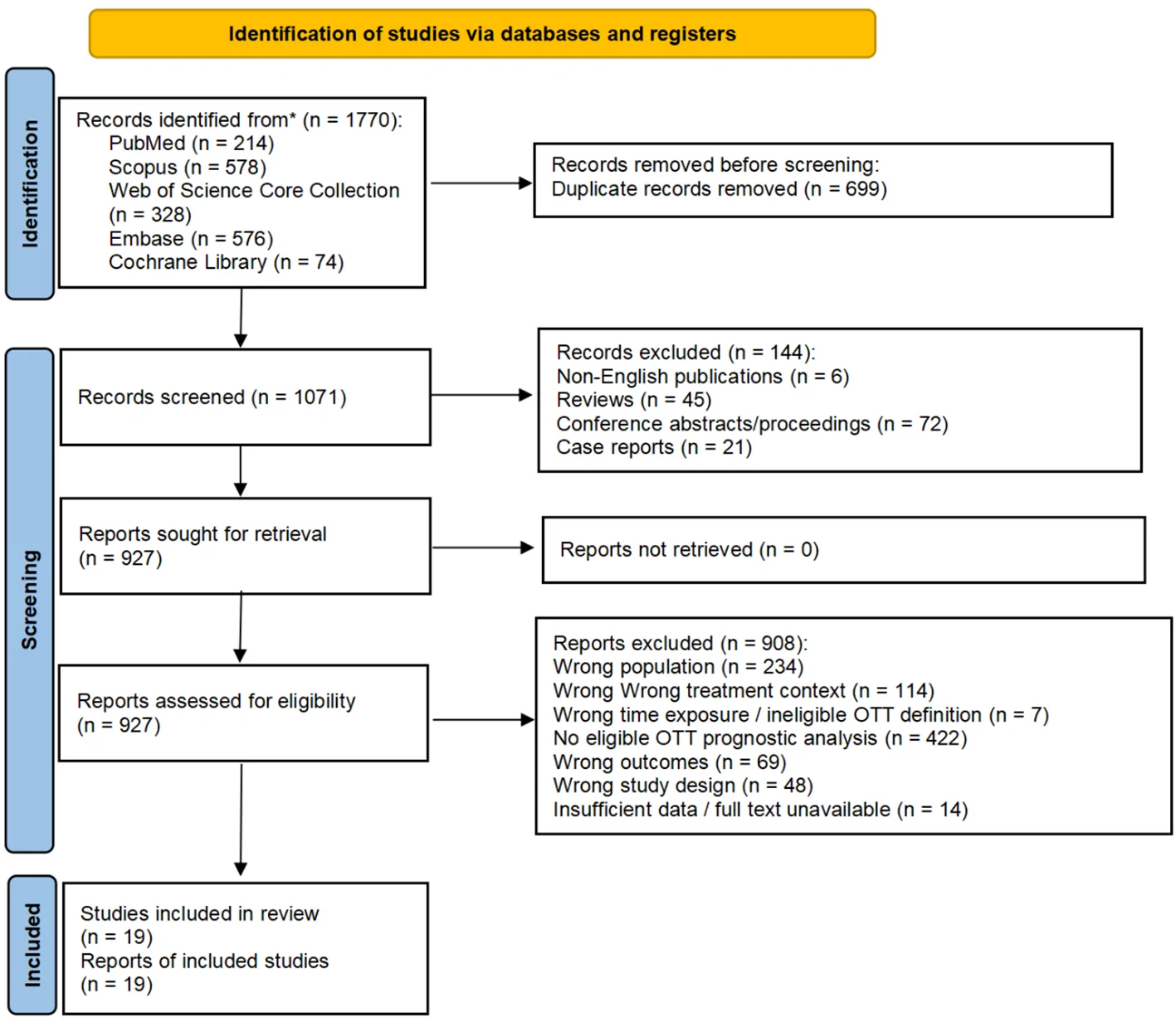
PRISMA 2020 flow diagram of study identification, screening, eligibility assessment, and inclusion. PRISMA, Preferred Reporting Items for Systematic Reviews and Meta-Analyses.

The 19 studies (10, 11, 16–32) included 15,859 patients and were published between 1993 and 2026. Most were retrospective observational cohorts (10, 11, 16–27, 29–32), including single- and multicenter institutional cohorts, national or registry-based analyses, and a Patterns-of-Care study (24); one was a secondary prognostic analysis of prospectively collected Gynecologic Oncology Group trial data (28). Study sample sizes ranged from 64 patients (23) to 7,355 patients (10). The evidence base included historical radiotherapy-dominant/radiotherapy-alone series, defined as cohorts in which radiotherapy without concurrent chemotherapy represented at least 50% of patients or no concurrent chemotherapy was reported (19–21, 24, 29) and more contemporary populations treated predominantly with concurrent chemoradiotherapy (10, 11, 16, 25, 27, 28, 30–32), with brachytherapy incorporated in most treatment settings. Studies varied considerably in treatment era, FIGO stage distribution, concurrent chemotherapy use, radiotherapy technique, and the OTT thresholds used to define treatment prolongation.

Study-specific OTT definitions also varied substantially. Categorical thresholds ranged from approximately 50 days to 80 days or longer (23, 26); some studies used multiple treatment-duration categories (24, 29), whereas others also modeled OTT as a continuous exposure (10, 11, 16, 20). Nevertheless, all included studies evaluated treatment duration within the definitive radiotherapy course in accordance with the prespecified eligibility framework. The main study characteristics are summarized in **Table 1**, detailed patient characteristics, treatment and OTT parameters, and extracted oncologic outcomes are provided in **Supplementary Tables S1**–**S3**.

**Table 1:** Main characteristics of the studies included in the systematic review and meta-analysis.

Study	Year	Country	Design	N	Age, years	FIGO stage	SCC, n (%)	Treatment setting	Concurrent chemotherapy, n (%)	Brachytherapy, n (%)	Follow-up	OTT, days	Primary OTT cutoff	Outcomes reported
Amneus et al. (16)	2015	USA	Retrospective, 2-center	128	Median 51 (25–90)	I: 22 (17.2); II: 64 (50.0); III: 39 (30.5); IVA: 3 (2.3)	107 (83.6)	Mixed RT/CCRT	120 (93.8)	128 (100.0)	Median 26 mo	Median 72	63	DFS; OS
Chatani et al. (17)	1997	Japan	Retrospective	216	33–83; age strata 101/115	IIB: 85 (39.4); III: 131 (60.6)	205 (94.9)	Definitive EBRT + HDR intracavitary irradiation	NR	216 (100.0)	Median 8.4 y (0.4–16.6)	NR	35–42 / 43–49 / 50–62 d	Local relapse / pelvic failure; Cause-specific survival
Chen et al. (18)	2003	Taiwan	Retrospective	257	Median 55 (32–87)	I: 35 (13.6); II: 148 (57.6); III: 67 (26.1); IVA: 7 (2.7)	252 (98.1)	RT alone ± adjuvant chemo	0 (0.0)	257 (100.0)	Median 57 mo (38–110)	Median 63	63	CSS; Pelvic failure
Delaloye et al. (19)	1997	Switzerland	Retrospective	360	Median 60 (20–90)	I: 21 (5.8); II: 182 (50.6); III: 157 (43.6)	327 (90.8)	Definitive EBRT + brachytherapy	NR	360 (100.0)	>12 y median follow-up	Median 45	60	OS
Erridge et al. (20)	2002	United Kingdom	Retrospective	647	Mean 54.4 ± 12.5 (24–79)	IB: 172 (26.6); IIA: 91 (14.1); IIB: 218 (33.7); IIIA: 9 (1.4); IIIB: 141 (21.8); remaining 16 NR†	592 (91.5)	RT alone	0 (0.0)	647 (100.0)	Minimum 7.5 y in 642; max 22 y	NR	29–32 / 33–38 / 39–44 / ≥45 d	OS; CSS; grade 4 late morbidity
Girinsky et al. (21)	1993	France	Retrospective	386	Mean 57.7	IIB: 115 (29.8); III: 271 (70.2)	354 (91.7)	RT alone	0 (0.0)	385 (99.7)	Outcome status updated to Jun 1992	Median 52	52	Local failure; OS
Hong et al. (10)	2017	USA	NCDB retrospective cohort	7355	Median 50	I: 1244 (16.9); II: 3798 (51.6); III: 2112 (28.7); IVA: 201 (2.7)	6073 (82.6)	Chemotherapy + EBRT + BT	Chemotherapy required; concurrent timing NR	7355 (100.0)	Median 33.6 mo in survivors	Median 57	64.9	OS
Horne et al. (11)	2019	USA	Single-institution retrospective	239	Median 53; IQR 43–64 (23–91)	IB1/IIA1: 28; IB2/IIA2: 23; IIB: 138; IIIB/IVA: 50	194 (81.2)	Definitive CRT/RT + MRI-HDR BT	NR	239 (100.0)	Median 28.6 mo (IQR 12.7–53.8)	Median 51	51	Local failure; distant failure; PFS; OS
Joseph et al. (22)	2020	Sri Lanka	Retrospective	113	Mean 58 (35–85)	IB: 7 (6.2); IIA: 3 (2.7); IIB: 52 (46.0); IIIA: 9 (8.0); IIIB: 40 (35.4); IVA: 2 (1.8)	73 (64.6)	Mixed RT/CCRT	103 (91.2)	113 (100.0)	Median 27 mo (5–60)†	Median 110	60	DFS
Kumar et al. (23)	2023	India	Retrospective	132	Median 50 (28–70)	IB2: 24 (18.2); IIA: 13 (9.8); IIB: 50 (37.9); III: 45 (34.1)	132 (100.0)	Mixed RT/CCRT	94 (71.2)	132 (100.0)	NR; irregular follow-up reported	Median 80	80	OS; Pelvic control
Lanciano et al. (24)	1993	USA	Patterns-of-Care retrospective	837	OTT-group means 53.1–56.9	I: 321 (38.4); II: 377 (45.0); III: 133 (15.9); stage NR: 6 (0.7)	837 (100.0)	Definitive EBRT + intracavitary RT	NR	837 (100.0)	Actuarial analyses to 4 y	NR	<6 / 6–7.9 / 8–9.9 / ≥10 wk	In-field pelvic recurrence; OS
Lin et al. (25)	2017	Taiwan	Nationwide registry cohort	2594	Mean ~62	IA: 12 (0.5); IB: 538 (20.7); IIA: 310 (12.0); IIB: 998 (38.5); IIIA: 92 (3.5); IIIB: 503 (19.4); IVA: 141 (5.4)	2,250 (86.7)	RT or CCRT	1832 (70.6)	2,217 (85.5)	NR	Median 59	56	CSS; OS
Mandal et al. (26)	2007	India	Retrospective	64	Median 45.5 (27–65)	IIB: 35 (54.7); IIIB: 29 (45.3)	64 (100.0)	RT alone	0 (0.0)	64 (100.0)	Median 37 mo (19–90)	NR	50	Local control; DFS
Mazeron et al. (27)	2015	France	Single-institution retrospective	225	Mean 48.5 ± 11.2	IB1: 6; IB2: 56; IIA: 18; IIB: 110; IIIA: 4; IIIB: 24; IVA: 7	189 (84.0)	CCRT predominant	213 (94.7)	225 (100.0)	Median 39 mo	Mean 50.9	55	Local failure
Monk et al. (28)	2007	USA	Secondary analysis, GOG trials	335	Median 47 (20–80)	IIB: 208 (62.1); IIIA: 3 (0.9); IIIB: 114 (34.0); IVA: 10 (3.0)	292 (87.2)	Protocol CCRT	335 (100.0)	Protocol-based; n NR	Median 75 mo (GOG120); 45 mo (GOG165)	Median GOG120: 60/65; GOG165: 51/53 by stage	GOG120: ≥10 wk; GOG165: ≥8 wk	PFS; OS
Perez et al. (29)	1995	USA	Retrospective	1224	NR	IB: 415 (33.9); IIA: 137 (11.2); IIB: 391 (31.9); III: 281 (23.0)	>90%	RT alone	0 (0.0)	1224 (100.0)	Median 12 y (3–28); 97% follow-up	NR	≤7 / 7.1–9 / >9 wk	Pelvic failure/control; DFS; CSS
Sahli et al. (30)	2016	Morocco	Single-institution retrospective	293	50 (44–59)†	IB: 28; IIA: 13; IIB: 131; IIIA: 2; IIIB: 114; IVA: 5	278 (94.9)	CCRT	293 (100.0)	71%	Median 31 mo	Median 61	56	OS; Local control / local failure
Shaverdian et al. (31)	2013	USA	Single-institution retrospective	372	<50: 193; ≥50: 179	IB: 112 (30.1); II: 163 (43.8); III–IVA: 97 (26.1)	317 (85.2)	RT 206 / CRT 166	166 (44.6)	372 (100.0)	Median relapse-free FU: RT 4.4 y; CRT 4.2 y	Median RT 55; CRT 51	No single cutoff; 49–63 d threshold sweep	DFS; OS; In-field relapse
Thasanthan et al. (32)	2026	Sri Lanka	Retrospective	82	Median 55 (25–73)	IIB: 41 (50.0); IIIA: 19 (23.2); IIIB: 14 (17.1); IVA: 8 (9.7)	57 (69.5)	CCRT	82 (100.0)	82 (100.0)	OS: median 36 mo (24–36; IQR 34–36); DFS: median 34 mo (12–36)	Median 75	75	OS; DFS

BT, brachytherapy; CCRT, concurrent chemoradiotherapy; FIGO, International Federation of Gynecology and Obstetrics; NR, not reported; OTT, overall treatment time; SCC, squamous cell carcinoma.

Values are shown as n (%) where available. † Source-internal discrepancies were retained in the audit trail; when a single value was required for tabulation, the most formal results table or Methods section was prioritized where possible: Amneus abstract n=129 vs Methods/**Table 1** n=128 (n=128 used); Chen abstract vs **Table 1** differs for IIIA/IIIB counts (**Table 1** used); Erridge stage counts reported in source **Table 1** sum to 631/647, with the remaining 16 not specified; Joseph abstract reports follow-up 28 mo whereas Results report 27 mo; Sahli Results report median age 49 y whereas **Table 1** reports 50 (44–59), and **Table 1** was used. Detailed patient variables, treatment parameters, OTT distributions, and effect estimates are provided in **Supplementary Tables S1**–**S3**.

### Risk of bias assessment

Using the QUIPS tool, 14 studies were judged to have moderate risk of bias (10, 11, 16–21, 25, 27–31), and five were judged to have high overall risk of bias (22–24, 26, 32) (**Table 2**). No study was judged to have low overall risk of bias.

**Table 2:** Risk of bias assessment using the Quality in Prognosis Studies (QUIPS) tool.

Study	Study participation	Study attrition	Prognostic factor measurement (OTT)	Outcome measurement	Study confounding	Statistical analysis and reporting	Overall risk of bias	Key rationale for judgment
Amneus et al. (16)	Moderate	Low	Low	Low	Moderate	Low	Moderate	Retrospective two-center cohort with selection based on treatment completion; OTT and outcomes were clearly measured, and multivariable adjustment was performed.
Chatani et al. (17)	Moderate	Low	Low	Low	Moderate	Low	Moderate	Retrospective dose-completer cohort; long follow-up; clear OTT categories and Cox models, but older single-center treatment and residual confounding remain.
Chen et al. (18)	Moderate	Low	Low	Low	Moderate	Low	Moderate	38/295 excluded for incomplete treatment/loss/bulky persistent disease; clear OTT and outcomes; stage and tumor response adjusted.
Delaloye et al. (19)	Moderate	Low	Low	Low	Moderate	Low	Moderate	360/612 selected for combined RT+BT; long follow-up and adjusted Cox analysis, but treatment-era/selection confounding persists.
Erridge et al. (20)	Moderate	Low	Low	Low	Moderate	Low	Moderate	Restricted to a standardized radical schedule; only 5 lost; OTT continuous and detailed confounders modeled, but historical treatment-selection effects remain.
Girinsky et al. (21)	Moderate	Low	Low	Low	Moderate	Low	Moderate	Homogeneous IIB–III RT-alone cohort with adjusted Cox models; residual confounding by tumor extent/boost and historical practice cannot be eliminated.
Hong et al. (10)	Low	Low	Low	Low	Moderate	Low	Moderate	Large NCDB cohort with robust validation and multivariable modeling; OS objective, but registry lacks granular treatment/interruption variables and is limited to accredited facilities.
Horne et al. (11)	Moderate	Low	Low	Low	Moderate	Low	Moderate	Single-institution dosimetric cohort; precise OTT/modern outcomes and Cox adjustment; selection of patients with evaluable MRI-guided BT and response-related confounding remain.
Joseph et al. (22)	High	Moderate	Low	Moderate	High	Moderate	High	Only 113/242 had complete analyzable treatment data; loss to follow-up treated as DFS event; delay effect remained univariable because no other factor reached entry criterion.
Kumar et al. (23)	High	High	Low	Moderate	High	High	High	Only 132/347 planned patients who completed protocol were included; authors acknowledge irregular follow-up and unmeasured confounding (e.g., anemia/comorbidity); no adjusted HR.
Lanciano et al. (24)	High	Moderate	Low	Low	Moderate	Moderate	High	837/1558 remained after extensive exclusions, including missing implant date and events before 4 months; adjusted significance reported but selection/time-bias concerns are substantial.
Lin et al. (25)	Low	Low	Low	Low	Moderate	Low	Moderate	Nationwide registry with large N and adjusted/propensity analyses; detailed nodal/interruption variables incomplete and treatment selection remains observational.
Mandal et al. (26)	High	Low	Low	Low	High	Moderate	High	Very small retrospective cohort (n=64); stage-stratified Kaplan–Meier and chi-square comparisons without multivariable adjustment or directly usable HRs.
Mazeron et al. (27)	Moderate	Low	Low	Low	Moderate	Low	Moderate	Modern single-center IGABT cohort with precise dosimetry and Cox adjustment; outcome-derived cutoff and response/dose confounding remain.
Monk et al. (28)	Low	Low	Low	Low	Moderate	Low	Moderate	Prospectively collected GOG trial data but OTT analysis was secondary observational; protocol/staging differences between GOG120 and GOG165 introduce heterogeneity.
Perez et al. (29)	Low	Low	Low	Low	Moderate	Low	Moderate	Large cohort with 97% long-term follow-up and stage/size/dose analyses; historical techniques and residual confounding by tumor burden remain.
Sahli et al. (30)	Moderate	Moderate	Low	Low	Moderate	Moderate	Moderate	Single-year retrospective completer cohort; multivariable Cox performed, but incomplete-treatment exclusion and reporting quality create some uncertainty.
Shaverdian et al. (31)	Moderate	Moderate	Low	Low	Low	Low	Moderate	372/480 evaluable after 58 lost to follow-up and 50 other exclusions; separate RT/CRT analyses, covariate adjustment and multiplicity correction strengthen analysis.
Thasanthan et al. (32)	High	Moderate	Low	Low	High	High	High	Small convenience sample (82/112) excludes incomplete treatment/progression and transfers; source contains internally inconsistent HR/CI reporting, limiting confidence in adjusted estimates.

QUIPS, Quality In Prognosis Studies. Domain-level ratings and overall judgments were based on reviewer assessment of information reported in each study. No numerical summary score was used; overall risk of bias reflected the methodological importance of the most consequential domains rather than simple arithmetic counting.

Prognostic factor measurement was generally judged to have low risk of bias because OTT was derived from documented radiotherapy dates and clearly defined in most studies. Outcome measurement was also generally robust, particularly for overall survival. The main methodological concerns involved study participation and selection, control of confounding, and statistical analysis or reporting. Several retrospective cohorts excluded substantial numbers of patients because of incomplete treatment, missing treatment dates, or incomplete follow-up, and some studies provided only unadjusted or minimally adjusted estimates. These concerns were most pronounced in studies judged to have high overall risk of bias (22–24, 26, 32). Detailed domain-level QUIPS judgments and supporting rationales are presented in **Table 2**.

### Primary outcomes

#### Overall survival

Ten independent cohorts (10, 11, 16, 19, 21, 25, 28, 30–32) contributed to the primary meta-analysis of OS. Longer OTT was associated with poorer OS (pooled HR, 1.61; 95% CI, 1.30–1.99; 95% prediction interval, 0.99–2.59; P < 0.0001) (**Figure 2**). Between-study heterogeneity was substantial (I² = 76%, P < 0.0001), suggesting considerable variability in the magnitude of the association across study settings.

**Figure 2:**
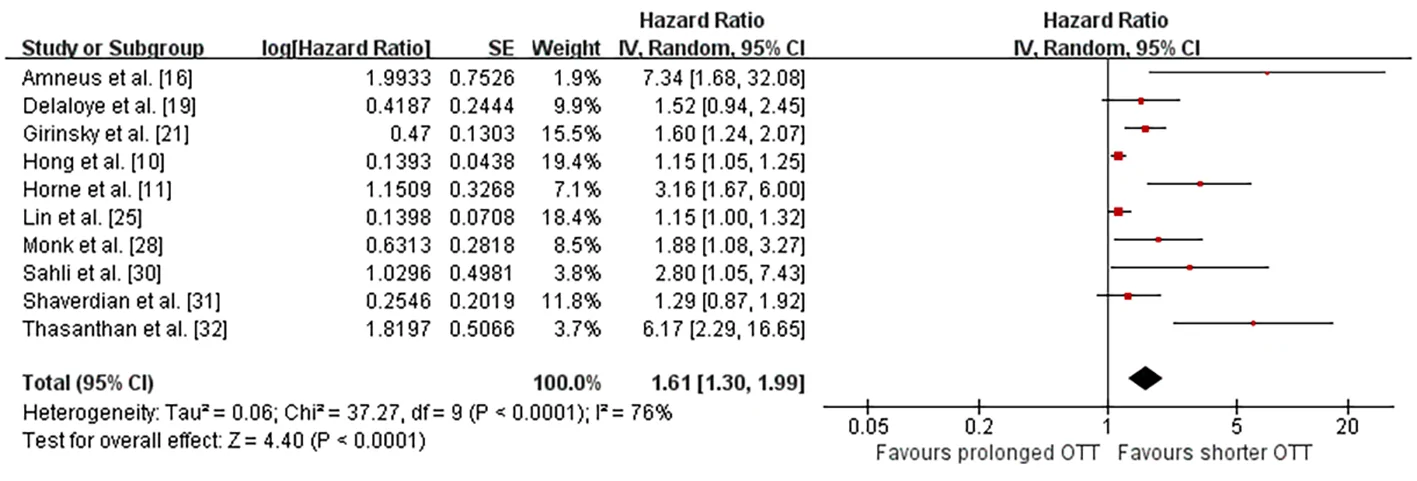
Forest plot of the random-effects meta-analysis evaluating the association between prolonged overall treatment time and overall survival. Hazard ratios greater than 1 indicate a higher risk of death with prolonged overall treatment time. CI, confidence interval; HR, hazard ratio; OTT, overall treatment time.

To evaluate the clinically established 8-week benchmark using a uniform exposure definition, a prespecified analysis was restricted to studies directly comparing OTT >56 days with ≤56 days. Three independent cohorts (10, 25, 30) met this strict definition. The association with poorer OS persisted beyond 56 days (HR, 1.16; 95% CI, 1.04–1.31; P = 0.01), with substantially lower heterogeneity than in the primary analysis (I² = 37%, P = 0.20) (**Figure 3**).

**Figure 3:**
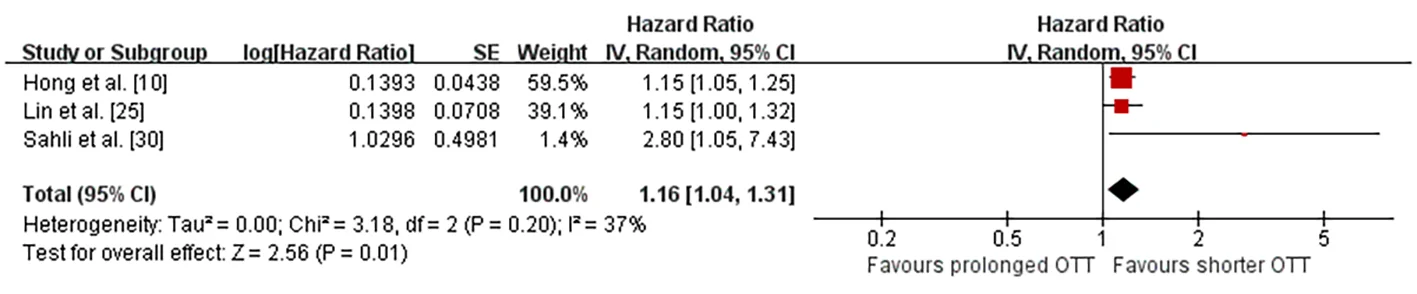
Forest plot of the random-effects meta-analysis evaluating overall survival using the prespecified uniform comparison of overall treatment time >56 versus ≤56 days. Hazard ratios greater than 1 indicate a higher risk of death with OTT >56 days. CI, confidence interval; HR, hazard ratio; OTT, overall treatment time.

#### Subgroup analyses of overall survival

In the prespecified subgroup analysis by treatment era and treatment mode, prolonged OTT was associated with poorer OS in historical radiotherapy-dominant or radiotherapy-alone cohorts (19, 21, 31) (HR, 1.50; 95% CI, 1.24–1.83; I² = 0%) and in modern chemotherapy-containing or concurrent chemoradiotherapy cohorts (10, 11, 16, 25, 28, 30, 32) (HR, 1.77; 95% CI, 1.32–2.38; I² = 81%) (**Figure 4**). There was no evidence of a difference between subgroups (P for subgroup difference = 0.36).

**Figure 4:**
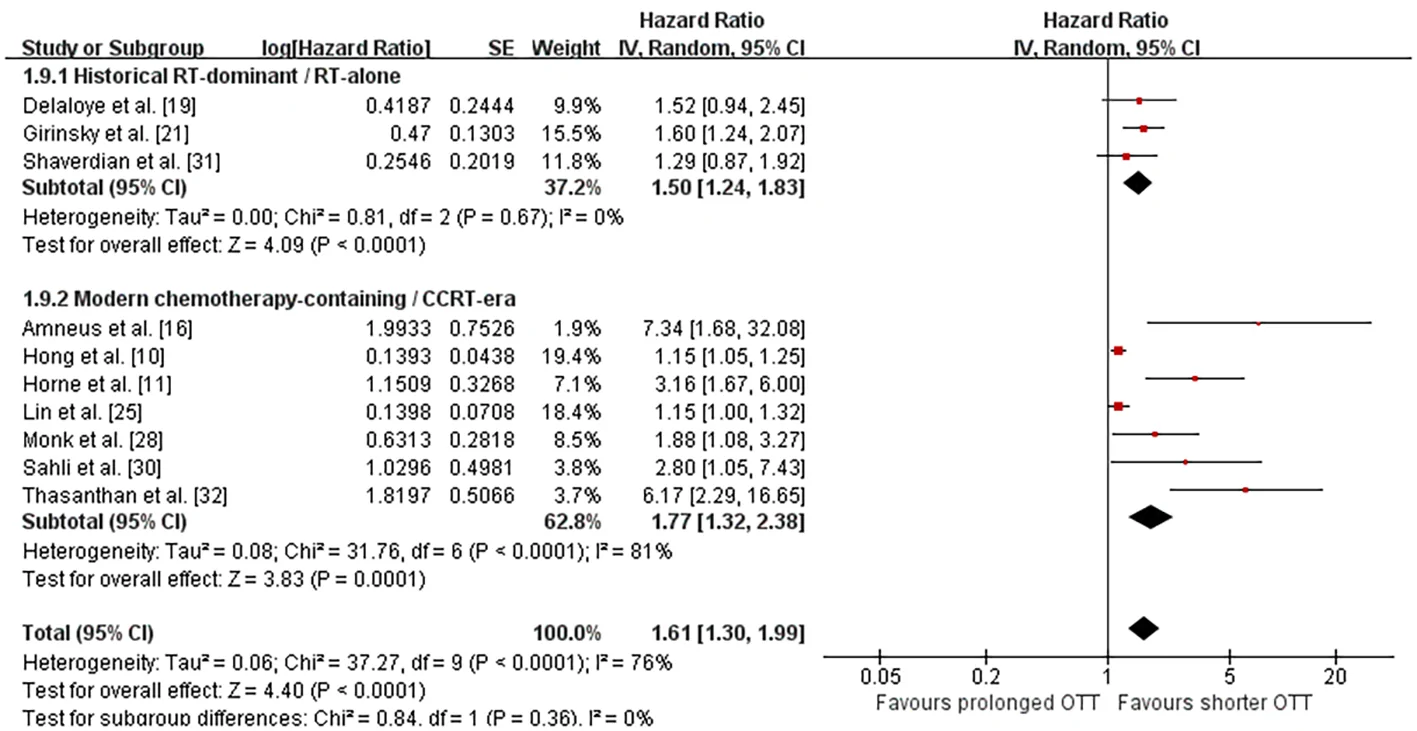
Forest plot of the subgroup analysis of overall survival according to treatment era and treatment mode. Treatment-era subgroups were classified according to the predominant treatment context reported in each original cohort, including historical radiotherapy-dominant/radiotherapy-alone and chemotherapy-containing/concurrent chemoradiotherapy cohorts. Hazard ratios greater than 1 indicate a higher risk of death with prolonged overall treatment time. CCRT, concurrent chemoradiotherapy; CI, confidence interval; HR, hazard ratio; OTT, overall treatment time; RT, radiotherapy.

When studies were stratified by the source of the OTT cutoff, the association remained significant among studies using clinical, guideline-based, or protocol-prespecified thresholds (10, 16, 25, 28, 30) (HR, 1.31; 95% CI, 1.06–1.62; I² = 67%) and among those using distribution-based, data-driven, or threshold-sweep cutoffs (11, 19, 21, 31, 32) (HR, 1.93; 95% CI, 1.32–2.80; I² = 68%) (**Figure 5**). Although the pooled estimate was numerically larger in the latter group, the subgroup difference was not statistically significant (P for subgroup difference = 0.08).

**Figure 5:**
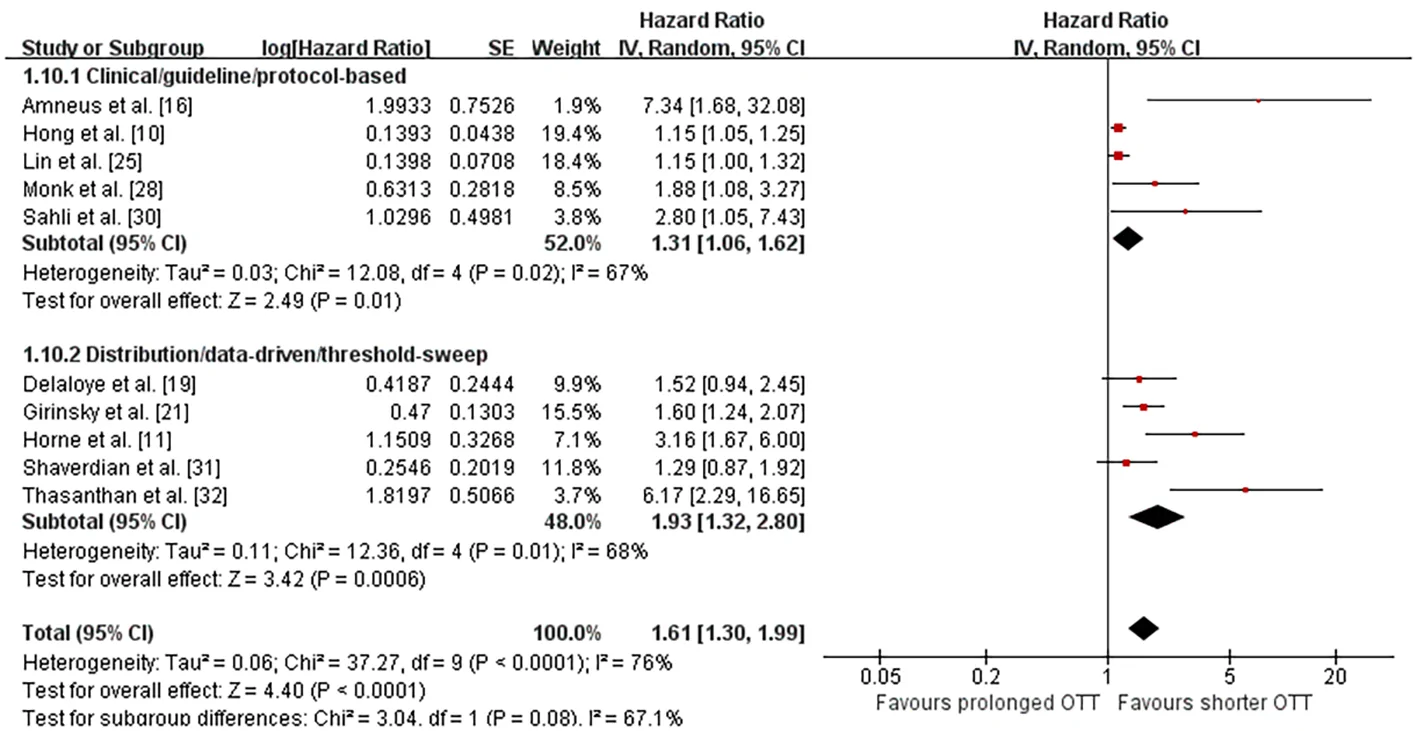
Forest plot of the subgroup analysis of overall survival according to the source of the overall treatment time cutoff. Studies were classified as using clinical, guideline-based, or protocol-prespecified thresholds versus distribution-based, data-driven, or threshold-sweep cutoffs. Hazard ratios greater than 1 indicate a higher risk of death with prolonged overall treatment time. CI, confidence interval; HR, hazard ratio; OTT, overall treatment time.

A further prespecified subgroup analysis by the magnitude of the study-specific OTT cutoff yielded a pooled HR of 1.42 (95% CI, 1.18–1.71; I² = 71%) for cutoffs ≤56 days (10, 11, 21, 25, 28, 30, 31). For cutoffs of 57–63 days (16, 19), the pooled HR was 2.84 (95% CI, 0.63–12.88; I² = 75%). The ≥64-day category was represented by a single study (32), with an HR of 6.17 (95% CI, 2.29–16.65) (**Figure 6**). This estimate was derived from a single study with a high risk of bias and should therefore not be interpreted as a precise or definitive estimate of the association. The test for subgroup differences was statistically significant (P = 0.01). However, the latter two strata contained only two studies and one study, respectively; this pattern should therefore be interpreted cautiously and not as evidence of a dose–response relationship between progressively longer cutoff values and mortality risk.

**Figure 6:**
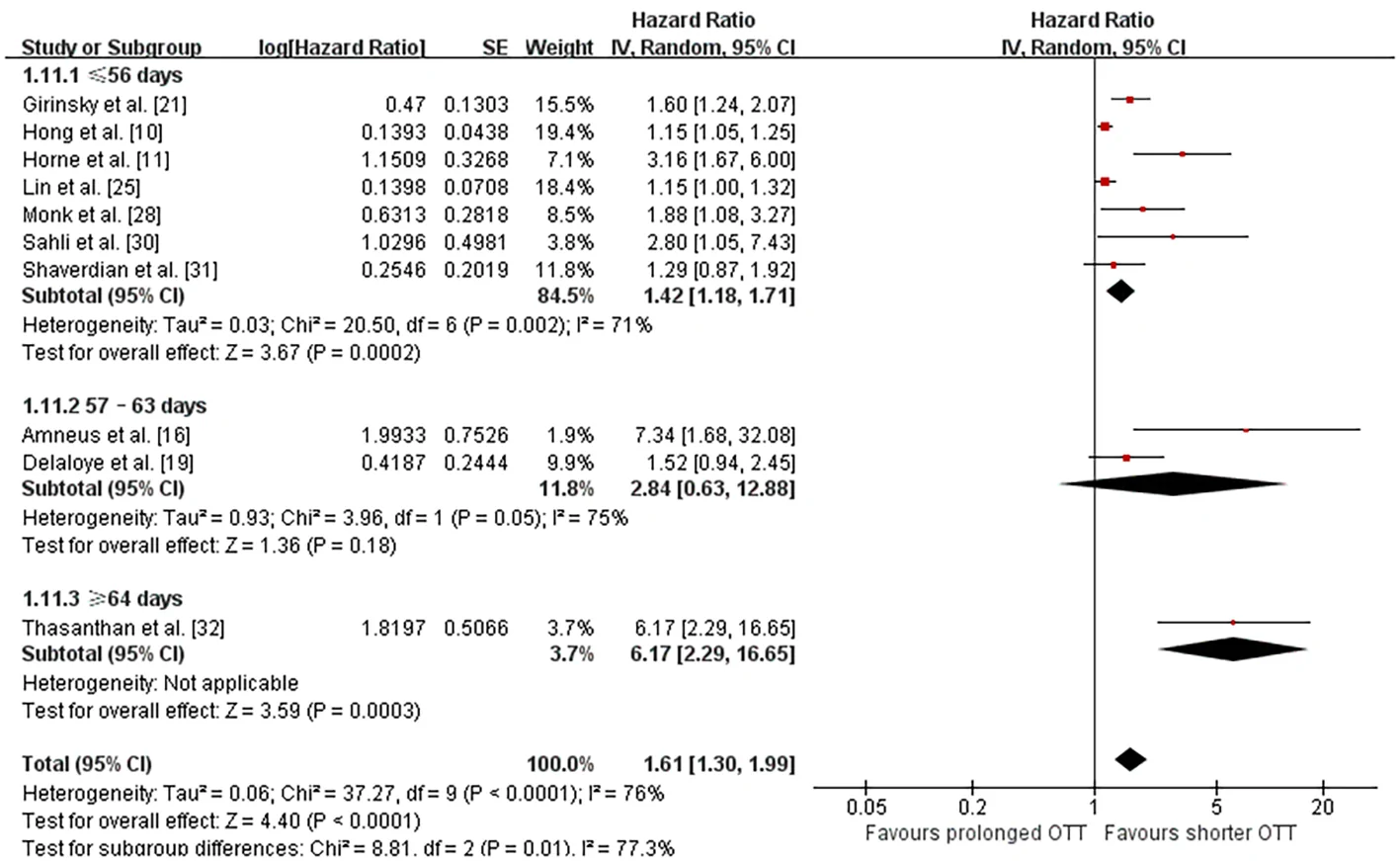
Forest plot of the subgroup analysis of overall survival according to the magnitude of the study-specific overall treatment time cutoff. Studies were grouped according to cutoffs of ≤56 days, 57–63 days, or ≥64 days. Hazard ratios greater than 1 indicate a higher risk of death with prolonged overall treatment time. CI, confidence interval; HR, hazard ratio; OTT, overall treatment time.

#### Sensitivity analyses of overall survival

The association between prolonged OTT and poorer OS remained robust across prespecified sensitivity analyses. Expanding the 56-day analysis to include studies using closely related 56-day threshold-family definitions (>56 vs ≤56 days or ≥56 vs <56 days) included five cohorts (10, 25, 28, 30, 31). The pooled association remained statistically significant (HR, 1.21; 95% CI, 1.06–1.37; P = 0.004), with moderate heterogeneity (I² = 37%, P = 0.17) (**Supplementary Figure S1**).

After exclusion of Thasanthan et al. (32), which was judged to have high overall risk of bias according to QUIPS, the association remained statistically significant (HR, 1.48; 95% CI, 1.23–1.80; P < 0.0001), although substantial heterogeneity persisted (I² = 70%, P = 0.0007) (**Supplementary Figure S2**).

Leave-one-out sensitivity analysis further supported the robustness of the primary OS finding. Sequential exclusion of each of the 10 contributing cohorts (10, 11, 16, 19, 21, 25, 28, 30–32) yielded pooled HRs ranging from 1.48 to 1.87, with 95% CIs remaining entirely above unity in every analysis. The lowest pooled estimate followed exclusion of Thasanthan et al. (32) (HR, 1.48; 95% CI, 1.23–1.80), whereas exclusion of Hong et al. (10) or Lin et al. (25) yielded the largest pooled estimates (HR, 1.87; 95% CI, 1.39–2.52 and HR, 1.87; 95% CI, 1.39–2.53, respectively). Heterogeneity remained substantial after sequential omission of individual studies (I² range, 70%–79%), indicating that no single study accounted for the observed between-study variability (**Supplementary Figure S3**).

The funnel plot for the primary OS analysis showed asymmetry, and Egger’s regression test indicated significant small-study effects (P < 0.001) (**Supplementary Figure S4**). Given the substantial between-study heterogeneity, this finding should not be interpreted as evidence of publication bias alone.

#### Local/pelvic/in-field failure

Five independent studies (18, 21, 27, 30, 31) contributed to the meta-analysis of local, pelvic, or in-field failure. Because individual studies used different definitions of locoregional failure-related outcomes, these events were analyzed collectively as reported by each study. Prolonged OTT was associated with a higher risk of local/pelvic/in-field failure (pooled HR, 1.80; 95% CI, 1.26–2.56; P = 0.001) (**Figure 7**). Between-study heterogeneity was moderate (I² = 46%, P = 0.11).

**Figure 7:**
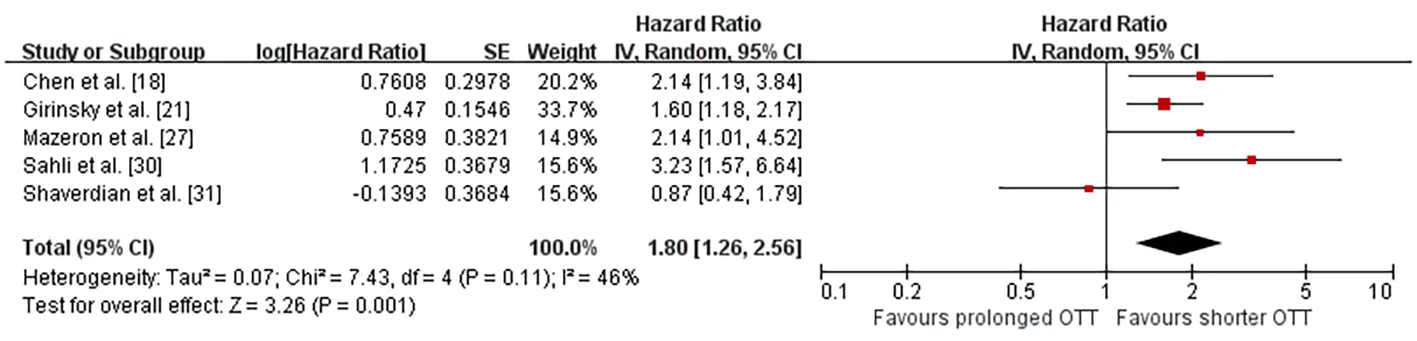
Forest plot of the random-effects meta-analysis evaluating the association between prolonged overall treatment time and local, pelvic, or in-field failure. Hazard ratios greater than 1 indicate a higher risk of failure with prolonged overall treatment time. CI, confidence interval; HR, hazard ratio; OTT, overall treatment time.

The certainty of evidence was rated as very low for the primary OS analysis and low for both the >56 versus ≤56-day OS analysis and the local/pelvic/in-field failure analysis. The main concerns included methodological limitations of the contributing studies, substantial heterogeneity in the primary OS analysis, the limited number of cohorts in the 56-day analysis, and variation in locoregional outcome definitions. Detailed judgments are provided in **Supplementary Table S4**.

#### Other secondary oncologic outcomes

Several additional oncologic outcomes were reported but could not be quantitatively synthesized because fewer than three independent studies provided clinically compatible estimates within the same outcome and adjustment stratum. For cause-specific/cancer-specific survival (CSS), adjusted effect estimates generally favored shorter OTT, with HRs/RRs ranging from 1.33 to 2.60 across studies (17, 18, 25). Adjusted DFS estimates were available from only two compatible cohorts, with HRs of 2.61 (95% CI, 1.22–5.56) and 1.42 (95% CI, 0.86–1.98) (16, 31). Three additional cohorts reported unadjusted DFS estimates (22, 31, 32), which were not combined with the adjusted estimates. Similarly, only two studies reported compatible adjusted PFS estimates (HR, 1.75; 95% CI, 0.95–3.32 and HR, 1.98; 95% CI, 1.16–3.38) (11, 28), and distant failure was evaluated in a single study (HR, 2.04; 95% CI, 0.99–4.20) (11).

Continuous OTT effects were reported in only a few studies. After standardization to a 7-day increment when numerical precision permitted, Amneus et al. (16) reported adjusted HRs of 1.09 (95% CI, 1.01–1.18) for OS and 1.04 (95% CI, 0.99–1.10) for DFS, whereas Erridge et al. (20) reported an adjusted HR of 0.98 (95% CI, 0.89–1.06) for CSS. In unadjusted analyses, Horne et al. (11) reported HRs per 7-day increase of 1.53 (95% CI, 1.28–1.84) for OS, 1.27 (95% CI, 1.04–1.56) for PFS, and 1.32 (95% CI, 1.04–1.68) for distant failure. Several other studies reported only fixed-time actuarial outcomes rather than compatible time-to-event effect estimates (23, 24, 26, 29), including progressively higher 4-year in-field pelvic recurrence with longer treatment duration in Lanciano et al. (24) and higher 10-year pelvic failure across longer OTT categories in Perez et al. (29). Because endpoint definition, OTT categorization, adjustment strategy, follow-up duration, and effect-measure availability differed across studies, these secondary outcomes were not formally pooled. Detailed study-specific results are provided in **Supplementary Table S5**.

## Discussion

In this systematic review and meta-analysis of 19 studies comprising 15,859 patients with cervical cancer treated with definitive radiotherapy or chemoradiotherapy, prolonged OTT was associated with poorer survival (HR, 1.61) and a higher risk of failure within the definitive treatment region (HR, 1.80). The OS association persisted in the uniform >56 versus ≤56-day analysis (HR, 1.16) and was evident in both historical radiotherapy-dominant and more contemporary chemotherapy-containing cohorts. These findings support the continued prognostic relevance of treatment duration, but not a simplistic “8-week rule.” Rather, the prognostic relevance of OTT appears to depend on how treatment prolongation is defined, the treatment context, and its underlying cause.

The adverse effect of prolonged treatment duration in cervical cancer has been recognized for decades. In the Patterns-of-Care analysis, Lanciano et al. reported an increase in 4-year in-field pelvic recurrence from 5.4% among patients completing treatment in <6 weeks to 19.9% among those treated for ≥10 weeks (24). Perez et al. likewise described worsening pelvic control with longer treatment durations (29), while Girinsky et al. and Delaloye et al. associated treatment prolongation with poorer local control or survival (19, 21). These studies established treatment duration as an important consideration in definitive radiotherapy, but they arose largely in an era of conventional EBRT and point-based intracavitary brachytherapy. Since then, concurrent chemotherapy, conformal and intensity-modulated EBRT, routine image guidance, and volumetric image-guided adaptive brachytherapy have substantially changed tumor dose delivery and normal-tissue sparing (33–35). The relevant question is therefore not simply whether the historical association can be reproduced, but whether treatment duration retains prognostic meaning across substantially different therapeutic eras. The persistence of an adverse association in chemotherapy-containing cohorts and the continued identification of OTT alongside tumor dose and volume in image-guided brachytherapy studies suggest that treatment advances have not rendered treatment time biologically irrelevant (7, 10, 11).

A time-dependent effect is biologically plausible because surviving clonogenic tumor cells may continue to proliferate during fractionated radiotherapy, and accelerated repopulation during a sufficiently prolonged course may partly offset the cytotoxic effect of the delivered radiation (36, 37). Cervical cancer–specific observations support this mechanism. Huang et al. estimated the onset of accelerated repopulation at approximately 19 days, although with considerable uncertainty, in patients treated with EBRT and low-dose-rate brachytherapy (38). In the image-guided brachytherapy era, Tanderup et al. estimated that approximately 5 Gy of additional HR-CTV D90 would be required to compensate for a 1-week increase in OTT for equivalent local control (7). Neither estimate defines a universal biological clock or dose-compensation rule, but together they indicate that treatment duration can influence the effective intensity of definitive irradiation. The locoregional meta-analysis provides complementary clinical support: across five independent studies (18, 21, 27, 30, 31), prolonged OTT was associated with a higher risk of local, pelvic, or in-field failure. These events are more proximal to the biological and technical effects of radiotherapy than OS, which is also influenced by distant progression, subsequent therapy, comorbidity, and competing mortality. Concordance across these endpoints strengthens the biological coherence of the association without establishing that locoregional failure mediates the observed survival effect. Because the pooled endpoint included local failure, pelvic failure, and in-field recurrence, it should be interpreted as failure within the definitive treatment region rather than specifically as recurrence of the primary cervical tumor.

The 56-day analysis is clinically useful but should not be overinterpreted. Restricting the analysis to studies using the exact comparison of >56 versus ≤56 days reduced variation in the definition of treatment prolongation and yielded a smaller, more homogeneous association with OS (10, 25, 30). Because the study sets differed, the smaller estimate does not indicate that prolongation beyond 56 days is biologically less important than prolongation defined by other thresholds. More fundamentally, OTT is a continuous exposure, and dichotomization makes the observed association dependent on the selected cutpoint; empirically selected thresholds may further exaggerate prognostic separation when several candidate cutoffs are examined (39–41). The sparse continuous data are therefore informative. Amneus et al. reported an adjusted OS HR of 1.09 per 7-day increase in OTT, whereas Horne et al. reported a larger unadjusted estimate of 1.53 per 7 days (11, 16); conversely, the adjusted continuous CSS estimate from Erridge et al. was close to null (20). These data are too sparse and heterogeneous to define a reliable per-week effect, but they reinforce an important point: the significant >56 versus ≤56-day association does not imply that risk changes abruptly on day 57. The 56-day interval is better regarded as an operationally useful benchmark than as a biologically validated boundary between “safe” and “unsafe” treatment durations. This interpretation is consistent with contemporary quality frameworks, which use treatment-completion targets as operational benchmarks rather than biologically validated boundaries (3, 9, 42).

Studies using data-derived or threshold-sweep cutoffs produced numerically larger estimates than those using clinical, guideline-based, or protocol-prespecified thresholds, although the subgroup interaction was not statistically significant. Likewise, progressively larger estimates across the ≤56-day, 57–63-day, and ≥64-day cutoff strata do not establish a dose–response relationship: the latter strata contained only two studies and one study, respectively, and the ≥64-day estimate came from a single cohort (32). These comparisons were made across different study populations that also varied in treatment era, case mix, radiotherapy technique, chemotherapy exposure, and brachytherapy practice. These differences likely contributed to the substantial heterogeneity in the primary OS analysis. The same factors likely contribute to the substantial heterogeneity in the primary OS analysis. Sensitivity analyses did not materially reduce this heterogeneity, and leave-one-out pooled HRs remained between 1.48 and 1.87. The evidence therefore supports a broadly adverse direction of association more strongly than any single estimate of its magnitude. The pooled HR is best interpreted as an average prognostic association across heterogeneous clinical settings rather than as the expected effect for every patient or treatment program.

These limitations are especially relevant to contemporary treatment. The absence of a significant interaction between historical radiotherapy-dominant and chemotherapy-containing cohorts provides no evidence that the prognostic relevance of OTT disappeared with the adoption of concurrent chemotherapy, it does not establish the same effect across treatment eras. “Modern CCRT” encompasses substantial variation in chemotherapy completion, EBRT technique, image guidance, brachytherapy technique and dose, disease burden, and institutional treatment pathways. Moreover, the included evidence does not adequately represent pembrolizumab-containing CCRT or induction chemotherapy followed by CCRT, and the findings should not be extrapolated directly to KEYNOTE-A18- or INTERLACE-type regimens (4–6). Treatment time should also not be considered in isolation from treatment completeness. Korenaga et al. provide an important counterpoint: among 10,172 women with stage II–IVA disease, only 29.3% both received brachytherapy and completed treatment within 8 weeks, yet among patients whose treatment exceeded 8 weeks, median OS was substantially longer in those who received brachytherapy than in those who did not (95.5 vs 49.2 months) (12). These observational data do not negate the adverse association with prolonged OTT; rather, they show that timeliness is one dimension of treatment quality, not a substitute for complete, technically adequate therapy. A calendar target should therefore not be achieved by omitting brachytherapy, reducing the indicated dose, or compromising target coverage.

This perspective also helps separate the potential biological effect of elapsed treatment time from the clinical processes that produce it. OTT may capture both a biological exposure—through continued clonogenic repopulation—and treatment complexity or care delivery. Longer treatment courses can result from toxicity, interruptions, equipment failure, delayed brachytherapy, interinstitutional referral, limited procedural capacity, or other barriers to completing planned therapy. Care fragmentation may also contribute to delay; in one analysis, treatment at different facilities was associated with more frequent delay than treatment at the same facility (43). This dual interpretation matters because not all prolonged courses represent the same prognostic pathway. Delay from fragmented referral or scheduling may be preventable, whereas prolongation associated with severe toxicity, extensive disease, or technically complex brachytherapy may also reflect underlying differences in baseline risk. Residual confounding by these factors is difficult to eliminate in retrospective prognostic studies and should temper causal interpretation of the pooled association.

The remaining secondary evidence is broadly consistent with this interpretation but is less definitive. Adjusted CSS estimates generally favored shorter OTT (17, 18, 25), and several DFS and PFS estimates were directionally unfavorable with longer treatment (11, 16, 28, 31). However, differences in endpoint definition, adjustment strategy, and the small number of compatible cohorts precluded reliable quantitative synthesis. These outcomes therefore provide triangulation rather than independent confirmation of the pooled OS and locoregional results. The inconsistency of secondary and continuous estimates also cautions against assuming a deterministic relationship between any single treatment duration and outcome.

Clinically, the findings do not justify enforcing a single binary OTT threshold at the expense of treatment quality. The priority is to identify and minimize avoidable prolongation while preserving treatment continuity, completeness, and technical quality. The 2026 AID-CARE quality-improvement initiative also suggests that treatment duration is partly modifiable: completion within 56 days increased from 18.6% to 70.1% after implementation of a bundled workflow (44). Early brachytherapy planning, allocation of procedural resources before completion of EBRT, proactive management of toxicity and treatment interruptions, clearly defined responsibility during interinstitutional referral, and prospective monitoring of the anticipated completion date are therefore reasonable clinical strategies. Future research should move beyond repeatedly deriving alternative “optimal” binary cutoffs. OTT should be defined consistently from the first definitive EBRT fraction through completion of all planned definitive radiotherapy, retained as a continuous exposure whenever possible, and modeled flexibly enough to detect nonlinear relationships when adequate data are available (45, 46). Future datasets should also record why treatment was prolonged and distinguish potentially modifiable delays from prolongation associated with disease burden, toxicity, or treatment complexity. Contemporary multicenter cohorts should incorporate current EBRT techniques, image-guided adaptive brachytherapy, brachytherapy dose and completion, chemotherapy delivery, and modern systemic-treatment strategies (4–6). The more informative question is no longer simply whether OTT matters, but how risk changes with additional prolongation, which mechanisms underlie the association, and which sources of delay can realistically be prevented.

Several features strengthen this review. The review prospectively restricted OTT to the definitive radiotherapy course, avoiding conflation with pretreatment waiting time, isolated interruptions, or component-specific intervals. Adjusted time-to-event estimates were prioritized, while categorical and continuous exposures, adjusted and unadjusted estimates, and incompatible effect measures were kept analytically separate. Study-specific definitions of prolonged OTT were evaluated separately from the uniform >56 versus ≤56-day comparison, allowing the overall prognostic association to be distinguished from the clinical utility of the conventional 8-week benchmark. Risk of bias was assessed using QUIPS, and subgroup and sensitivity analyses examined treatment era, cutoff definition, risk of bias, and individual-study influence. Quantitative synthesis was restricted to clinically compatible independent cohorts rather than undertaken solely to increase the number of pooled endpoints.

Several limitations constrain inference. Most included studies were retrospective and observational, and treatment duration was not randomly assigned. Patients with longer OTT may differ in disease burden, toxicity, treatment complexity, treatment completion, or access to care; residual confounding therefore remains possible despite preferential use of adjusted estimates. The evidence base also spans more than three decades of evolving radiotherapy and systemic treatment; 9 of the 19 included studies (47.4%) were published before 2010, and only one of the 10 cohorts contributing to the primary OS meta-analysis (10%) was published within the past five years. Studies also varied markedly in patient characteristics, brachytherapy practice, OTT definition, and cutoff selection. Individual studies have suggested that disease stage may influence the magnitude of the association between OTT and oncologic outcomes; for example, Lin et al. reported that the association between OTT and survival differed across stage groups (25). However, whether stage modifies the prognostic effect of OTT remains uncertain. Five studies were judged to have high overall risk of bias using QUIPS, and the certainty of evidence was very low for the primary OS analysis and low for the 56-day and locoregional analyses. The primary pooled estimate should therefore not be interpreted as a universal or precise estimate of risk in contemporary practice. Data were particularly sparse for longer cutoff categories and continuous OTT, limiting characterization of the underlying exposure–response relationship. Although FIGO stage was extracted for all included studies, stage-stratified quantitative analyses were not performed because stage-specific effect estimates were not consistently reported, and stage distributions and eligibility criteria varied substantially across cohorts, precluding a reliable pooled stage-stratified analysis. Whether stage modifies the prognostic effect of OTT therefore requires confirmation in larger, harmonized datasets. Locoregional and other secondary outcomes were reported less consistently than OS, and differences in endpoint definitions and analytical adjustment prevented several potentially informative syntheses. Restriction to peer-reviewed English-language full-text reports may have introduced selection related to language or publication status, and some relevant studies may not have been identified despite searching multiple databases, screening reference lists, and performing forward citation tracking. In addition, significant funnel-plot asymmetry was observed for the primary OS analysis, although its interpretation is complicated by substantial clinical and methodological heterogeneity. These limitations mean that the results support a prognostic association but do not establish that each additional day of treatment delay directly causes a corresponding increase in mortality risk.

In conclusion, prolonged OTT remains an adverse prognostic marker in cervical cancer across substantially different treatment settings. Clinical interpretation should emphasize timely and complete delivery of definitive radiotherapy, particularly brachytherapy, rather than rigid adherence to a single cutoff. The 56-day interval remains a clinically useful benchmark but should not be regarded as a biologically fixed boundary. Future studies should better define the continuous exposure–response relationship and distinguish the prognostic consequences of different causes of treatment prolongation.

## Data Availability

The original contributions presented in the study are included in the article/**Supplementary Material**. Further inquiries can be directed to the corresponding author.

## Supplementary material

Supplementary Figure 1 Sensitivity analysis of overall survival using the expanded 56-day threshold-family definition, including studies comparing overall treatment time >56 versus ≤56 days or ≥56 versus <56 days. The analysis is presented as a random-effects forest plot. Hazard ratios greater than 1 indicate a higher risk of death with prolonged overall treatment time. CI, confidence interval; HR, hazard ratio; OTT, overall treatment time.

Supplementary Figure 2 Sensitivity analysis of overall survival after exclusion of Thasanthan et al., which was judged to have high overall risk of bias according to the Quality In Prognosis Studies assessment. The analysis is presented as a random-effects forest plot. Hazard ratios greater than 1 indicate a higher risk of death with prolonged overall treatment time. CI, confidence interval; HR, hazard ratio; OTT, overall treatment time; QUIPS, Quality In Prognosis Studies.

Supplementary Figure 3 Leave-one-out sensitivity analysis of the primary overall survival meta-analysis. Each row shows the pooled hazard ratio and 95% confidence interval after sequential exclusion of one contributing study; the corresponding I² value is also shown. The main pooled estimate before study exclusion was HR 1.61 (95% CI, 1.30–1.99). CI, confidence interval; HR, hazard ratio; OTT, overall treatment time.

Supplementary Figure 4 Funnel plot for the primary overall survival meta-analysis assessing potential small-study effects. Each point represents one independent cohort included in the primary OS synthesis.

## References

[1] Sung H Filho AM Laversanne M Ferlay J Siegel RL Soerjomataram I . Global cancer statistics 2024: GLOBOCAN estimates of incidence and mortality worldwide for 34 cancers in 186 countries. CA Cancer J Clin. (2026) 76:e70090. doi: 10.3322/caac.70090 42417444 PMC13343830

[2] Chino J Annunziata CM Beriwal S Bradfield L Erickson BA Fields EC . Radiation therapy for cervical cancer: Executive summary of an ASTRO clinical practice guideline. Pract Radiat Oncol. (2020) 10:220–34. doi: 10.1016/j.prro.2020.04.002 32473857 PMC8802172

[3] Cibula D Rosaria Raspollini M Planchamp F Centeno C Chargari C Felix A . ESGO/ESTRO/ESP guidelines for the management of patients with cervical cancer - Update 2023. Radiother Oncol. (2023) 184:109682. doi: 10.1016/j.radonc.2023.109682 37336614

[4] Pötter R Tanderup K Schmid MP Jürgenliemk-Schulz I Haie-Meder C Fokdal LU . MRI-guided adaptive brachytherapy in locally advanced cervical cancer (EMBRACE-I): a multicentre prospective cohort study. Lancet Oncol. (2021) 22:538–47. doi: 10.1016/S1470-2045(20)30753-1 33794207

[5] Lorusso D Xiang Y Hasegawa K Scambia G Leiva M Ramos-Elias P . Pembrolizumab or placebo with chemoradiotherapy followed by pembrolizumab or placebo for newly diagnosed, high-risk, locally advanced cervical cancer (ENGOT-cx11/GOG-3047/KEYNOTE-A18): overall survival results from a randomised, double-blind, placebo-controlled, phase 3 trial. Lancet. (2024) 404:1321–32. doi: 10.1016/S0140-6736(24)01808-7 39288779

[6] McCormack M Eminowicz G Gallardo D Diez P Farrelly L Kent C . Induction chemotherapy followed by standard chemoradiotherapy versus standard chemoradiotherapy alone in patients with locally advanced cervical cancer (GCIG INTERLACE): an international, multicentre, randomised phase 3 trial. Lancet. (2024) 404:1525–35. doi: 10.1016/S0140-6736(24)01438-7 39419054

[7] Tanderup K Fokdal LU Sturdza A Haie-Meder C Mazeron R van Limbergen E . Effect of tumor dose, volume and overall treatment time on local control after radiochemotherapy including MRI guided brachytherapy of locally advanced cervical cancer. Radiother Oncol. (2016) 120:441–6. doi: 10.1016/j.radonc.2016.05.014 27350396

[8] González Ferreira JA Fernandez C Gonsalves D Paguey I Couñago F . Radiotherapy treatment time delay evidence, part I: Update on cervical, anal, prostate, and head and neck cancers. World J Clin Oncol. (2025) 16:109247. doi: 10.5306/wjco.v16.i10.109247 41178919 PMC12576527

[9] Chargari C Tanderup K Planchamp F Chiva L Humphrey P Sturdza A . ESGO/ESTRO quality indicators for radiation therapy of cervical cancer. Radiother Oncol. (2023) 183:109589. doi: 10.1016/j.radonc.2023.109589 37268359

[10] Hong JC Foote J Broadwater G Sosa JA Gaillard S Havrilesky LJ . Data-derived treatment duration goal for cervical cancer: Should 8 weeks remain the target in the era of concurrent chemoradiation? JCO Clin Cancer Inform. (2017) 1:1–5. doi: 10.1200/CCI.16.00072 30657372

[11] Horne ZD Karukonda P Kalash R Edwards RP Kelley JL Comerci JT . Single-institution experience in 3D MRI-based brachytherapy for cervical cancer for 239 women: Can dose overcome poor response? Int J Radiat Oncol Biol Phys. (2019) 104:157–64. doi: 10.1016/j.ijrobp.2018.12.042 30605752

[12] Korenaga TK Yoshida EJ Pierson W Chang J Ziogas A Swanson ML . Better late than never: Brachytherapy is more important than timing in treatment of locally advanced cervical cancer. Gynecol Oncol. (2022) 164:348–56. doi: 10.1016/j.ygyno.2021.11.015 34865860

[13] Page MJ McKenzie JE Bossuyt PM Boutron I Hofmann TC Mulrow CD . The PRISMA 2020 statement: An updated guideline for reporting systematic reviews. BMJ. (2021) 372:n71. doi: 10.1136/bmj.n71 33782057 PMC8005924

[14] Higgins JPT Thomas J Chandler J Cumpston M Li T Page MJ . Cochrane Handbook for Systematic Reviews of Interventions Version 6.3 (Updated February 2022) (2022). Chichester: Cochrane. Available online at: https://training.cochrane.org/handbook/current (Accessed August 3, 2026).

[15] Hayden JA van der Windt DA Cartwright JL Côté P Bombardier C . Assessing bias in studies of prognostic factors. Ann Intern Med. (2013) 158:280–6. doi: 10.7326/0003-4819-158-4-201302190-00009 23420236

[16] Amneus MW Park S Delic L Chung P Botnick M Cass I . Survival impact of prolonged treatment duration in primary chemoradiation for cervical cancer. Obstet Gynecol Int J. (2015) 3:314–21. doi: 10.15406/ogij.2015.03.00081

[17] Chatani M Matayoshi Y Masaki N Inoue T . High-dose rate intracavitary irradiation for carcinoma of the uterine cervix. The adverse effect of treatment prolongation. Strahlenther Onkol. (1997) 173:379–84. doi: 10.1007/BF03038241 9236934

[18] Chen SW Liang JA Yang SN Ko HL Lin FJ . The adverse effect of treatment prolongation in cervical cancer by high-dose-rate intracavitary brachytherapy. Radiother Oncol. (2003) 67:69–76. doi: 10.1016/s0167-8140(02)00439-5 12758242

[19] Delaloye JF Coucke PA Pampallona S Peltecu G De Grandi P . Radiation therapy duration influences overall survival in patients with cervical carcinoma. Int J Gynaecol Obstet. (1997) 57:295–303. doi: 10.1016/s0020-7292(97)02888-9 9215493

[20] Erridge SC Kerr GR Downing D Duncan W Price A . The effect of overall treatment time on the survival and toxicity of radical radiotherapy for cervical carcinoma. Radiother Oncol. (2002) 63:59–66. doi: 10.1016/s0167-8140(02)00012-9 12065104

[21] Girinsky T Rey A Roche B Haie C Gerbaulet A Randrianarivello H . Overall treatment time in advanced cervical carcinomas: A critical parameter in treatment outcome. Int J Radiat Oncol Biol Phys. (1993) 27:1051–6. doi: 10.1016/0360-3016(93)90522-w 8262826

[22] Joseph N Jayalath H Balawardena J Skandarajah T Perera K Gunasekera D . Radical external-beam radiotherapy in combination with intracavitary brachytherapy for localized carcinoma of the cervix in Sri Lanka: Is treatment delayed treatment denied? JCO Glob Oncol. (2020) 6:1574–81. doi: 10.1200/GO.20.00196 33079608 PMC7605371

[23] Kumar MN Kumar AS Srinivasan V . Retrospective analysis on the impact of treatment time on survival in squamous cell carcinoma of cervix. Int J Acad Med Pharm. (2023) 5:173–7.

[24] Lanciano RM Pajak TF Martz K Hanks GE . The influence of treatment time on outcome for squamous cell cancer of the uterine cervix treated with radiation: A patterns-of-care study. Int J Radiat Oncol Biol Phys. (1993) 25:391–7. doi: 10.1016/0360-3016(93)90058-4 8436516

[25] Lin SM Ku HY Chang TC Liu TW Hong JH . The prognostic impact of overall treatment time on disease outcome in uterine cervical cancer patients treated primarily with concomitant chemoradiotherapy: A nationwide Taiwanese cohort study. Oncotarget. (2017) 8:85203–13. doi: 10.18632/oncotarget.19617 29156713 PMC5689603

[26] Mandal A Asthana AK Aggarwal LM . Clinical significance of cumulative biological effective dose and overall treatment time in the treatment of carcinoma cervix. J Med Phys. (2007) 32:68–72. doi: 10.4103/0971-6203.33244 21157538 PMC3000534

[27] Mazeron R Castelnau-Marchand P Dumas I del Campo ER Kom LK Martinetti F . Impact of treatment time and dose escalation on local control in locally advanced cervical cancer treated by chemoradiation and image-guided pulsed-dose rate adaptive brachytherapy. Radiother Oncol. (2015) 114:257–63. doi: 10.1016/j.radonc.2014.11.045 25497872

[28] Monk BJ Tian C Rose PG Lanciano R . Which clinical/pathologic factors matter in the era of chemoradiation as treatment for locally advanced cervical carcinoma? Analysis of two Gynecologic Oncology Group (GOG) trials. Gynecol Oncol. (2007) 105:427–33. doi: 10.1016/j.ygyno.2006.12.027 17275889 PMC1940233

[29] Perez CA Grigsby PW Castro-Vita H Lockett MA . Carcinoma of the uterine cervix. I. Impact of prolongation of overall treatment time and timing of brachytherapy on outcome of radiation therapy. Int J Radiat Oncol Biol Phys. (1995) 32:1275–88. doi: 10.1016/0360-3016(95)00220-S 7635767

[30] Sahli N Khalil J Yatribi K Mouzount A Elkacemi H Elmajjaoui S . Impact of treatment duration on cervical cancer outcomes: Results from a single institution. Gynecol Obstet (Sunnyvale). (2016) 6:354. doi: 10.4172/2161-0932.1000354

[31] Shaverdian N Gondi V Sklenar KL Dunn EF Petereit DG Straub MR . Effects of treatment duration during concomitant chemoradiation therapy for cervical cancer. Int J Radiat Oncol Biol Phys. (2013) 86:562–8. doi: 10.1016/j.ijrobp.2013.01.037 23561652

[32] Thasanthan L Hilmi AJ Rosairo S Abeysundara HTK . Impact of treatment duration on cervical cancer outcomes with concomitant chemoradiation. Radiography (Lond). (2026) 32:103375. doi: 10.1016/j.radi.2026.103375 41780157

[33] Cohen PA Jhingran A Oaknin A Denny L . Cervical cancer. Lancet. (2019) 393:169–82. doi: 10.1016/S0140-6736(18)32470-X 30638582

[34] Pötter R Tanderup K Kirisits C de Leeuw A Kirchheiner K Nout R . The EMBRACE II study: The outcome and prospect of two decades of evolution within the GEC-ESTRO GYN working group and the EMBRACE studies. Clin Transl Radiat Oncol. (2018) 9:48–60. doi: 10.1016/j.ctro.2018.01.001 29594251 PMC5862686

[35] Williamson CW Liu HC Mayadev J Mell LK . Advances in external beam radiation therapy and brachytherapy for cervical cancer. Clin Oncol (R Coll Radiol). (2021) 33:567–78. doi: 10.1016/j.clon.2021.06.012 34266728

[36] Withers HR Taylor JM Maciejewski B . The hazard of accelerated tumor clonogen repopulation during radiotherapy. Acta Oncol. (1988) 27:131–46. doi: 10.3109/02841868809090333 3390344

[37] Baumann M Krause M Hill R . Exploring the role of cancer stem cells in radioresistance. Nat Rev Cancer. (2008) 8:545–6. doi: 10.1038/nrc2419 18511937

[38] Huang Z Mayr NA Gao M Lo SS Wang JZ Jia G . Onset time of tumor repopulation for cervical cancer: First evidence from clinical data. Int J Radiat Oncol Biol Phys. (2012) 84:478–84. doi: 10.1016/j.ijrobp.2011.12.037 22386374 PMC3369115

[39] Riley RD Moons KGM Snell KIE Ensor J Hooft L Altman DG . A guide to systematic review and meta-analysis of prognostic factor studies. BMJ. (2019) 364:k4597. doi: 10.1136/bmj.k4597 30700442

[40] Altman DG Royston P . The cost of dichotomising continuous variables. BMJ. (2006) 332:1080. doi: 10.1136/bmj.332.7549.1080 16675816 PMC1458573

[41] Altman DG Lausen B Sauerbrei W Schumacher M . Dangers of using "optimal" cutpoints in the evaluation of prognostic factors. J Natl Cancer Inst. (1994) 86:829–35. doi: 10.1093/jnci/86.11.829 8182763

[42] Neibart SS Lin LL Einstein MH Teoh D Leath CA Chino J . Minimum standards for radiation therapy in the treatment of cervical cancer in the U.S.: A consensus statement by SGO, ASTRO, and ABS addressing the WHO Cervical Cancer Elimination Campaign goals. Gynecol Oncol. (2025) 200:180–5. doi: 10.1016/j.ygyno.2025.07.028 40947173

[43] Li R Germino E Horne ZD Vargo JA Chen YJ Glaser S . External beam radiation and brachytherapy boost at different facilities is associated with increased treatment delays in cervical cancer. Int J Gynecol Cancer. (2020) 30:1505–12. doi: 10.1136/ijgc-2020-001700 32928924

[44] Bonala SR Subramanian BV Venati C Selvamary H Talatoti NB Sindhu G . Improving compliance with curative radiotherapy for cervical cancer in a high-volume centre in India: A bundled workflow (AID-CARE) quality improvement project. BMJ Open Qual. (2026) 15:e003874. doi: 10.1136/bmjoq-2025-003874 42409422 PMC13343099

[45] Discacciati A Palazzolo MG Park JG Melloni GEM Murphy SA Bellavia A . Estimating and presenting non-linear associations with restricted cubic splines. Int J Epidemiol. (2025) 54:dyaf088. doi: 10.1093/ije/dyaf088 40527479

[46] Crippa A Discacciati A Bottai M Spiegelman D Orsini N . One-stage dose-response meta-analysis for aggregated data. Stat Methods Med Res. (2019) 28:1579–96. doi: 10.1177/0962280218773122 29742975

