# Supplementary material for: Overall treatment time and oncologic outcomes after definitive radiotherapy or chemoradiotherapy for cervical cancer: a systematic review and meta-analysis
Source: Front Oncol. 2026 Oct 26;16:1969525. doi: 10.3389/fonc.2026.1969525 (PMC13745232; doi:10.3389/fonc.2026.1969525)
Supplement: Supplementary file 1 [file Table5.docx]

**Supplementary Methods: Literature search strategy**

We systematically searched PubMed/MEDLINE, Embase, Web of Science Core Collection, Scopus, and the Cochrane Library to identify original clinical studies evaluating the association between overall treatment time and oncologic outcomes in patients with cervical cancer treated with definitive radiotherapy or chemoradiotherapy. Searches covered each database from inception to 1 August 2026. The reference lists of included studies and relevant reviews or guidelines were manually screened, and forward citation tracking was used to identify additional eligible reports.

The fixed electronic search combined four mandatory concept groups: cervical cancer; radiotherapy, chemoradiotherapy, or brachytherapy; overall treatment time, treatment duration, treatment prolongation, interruption, breaks, or gaps; and oncologic outcomes or prognosis. The displayed strategies contain free-text terms only. Controlled-vocabulary terms, including MeSH and Emtree headings, were not included in the written strategies because they were selected separately within the relevant database interfaces. Broad low-specificity expressions related to elapsed time, generic treatment completion, and unrestricted clinical outcomes were excluded to reduce retrieval of studies concerning pretreatment waiting time, workflow duration, treatment adherence, or technical procedure time. Individual cutoff values such as 52, 55, 56, 63, or 65 days, disease stage, age, histologic subtype, effect measures, and study-design filters were not used as mandatory concepts. No publication-type NOT filter was applied. Database-specific field selection, wrappers, quotation marks, and punctuation were adapted to each platform; the four conceptual sets remained fixed.

**1. PubMed/MEDLINE**

**Database:** PubMed/MEDLINE

**Date range:** from database inception to 1 August 2026

**Search field:** Title/Abstract

**Search strategy:**

("cervical cancer" OR "cervical carcinoma" OR "cancer of the cervix" OR "carcinoma of the cervix" OR "uterine cervical cancer" OR "uterine cervical carcinoma")

AND

(radiotherap* OR "radiation therapy" OR chemoradiotherap* OR chemoradiation OR radiochemotherap* OR "concurrent chemoradiotherapy" OR "concurrent chemoradiation" OR CCRT OR brachytherap* OR "intracavitary radiation" OR "intracavitary radiotherapy" OR "intracavitary irradiation" OR "external beam radiotherapy" OR "external beam radiation therapy" OR EBRT)

AND

("overall treatment time" OR "overall treatment times" OR "overall treatment duration" OR "total treatment time" OR "total treatment duration" OR "treatment time" OR "treatment duration" OR "prolonged overall treatment time" OR "prolonged treatment time" OR "prolonged treatment duration" OR "protracted treatment" OR "treatment prolongation" OR "treatment protraction" OR "treatment interruption" OR "treatment interruptions" OR "radiotherapy interruption" OR "radiotherapy interruptions" OR "radiation interruption" OR "radiation interruptions" OR "treatment break" OR "treatment breaks" OR "radiotherapy break" OR "radiotherapy breaks" OR "treatment gap" OR "treatment gaps")

AND

("oncologic outcome" OR "oncologic outcomes" OR "oncological outcome" OR "oncological outcomes" OR prognos* OR survival OR "overall survival" OR "cancer-specific survival" OR "disease-specific survival" OR "disease-free survival" OR "progression-free survival" OR "recurrence-free survival" OR recurren* OR "local control" OR "pelvic control" OR "locoregional control" OR "tumor control" OR "tumour control" OR "local failure" OR "pelvic failure" OR "locoregional failure" OR "distant failure")

**2. Embase**

**Database:** Embase

**Date range:** from database inception to 1 August 2026

**Search field:** Title/Abstract/Author keywords

**Search strategy:**

('cervical cancer' OR 'cervical carcinoma' OR 'cancer of the cervix' OR 'carcinoma of the cervix' OR 'uterine cervical cancer' OR 'uterine cervical carcinoma')

AND

(radiotherap* OR 'radiation therapy' OR chemoradiotherap* OR chemoradiation OR radiochemotherap* OR 'concurrent chemoradiotherapy' OR 'concurrent chemoradiation' OR CCRT OR brachytherap* OR 'intracavitary radiation' OR 'intracavitary radiotherapy' OR 'intracavitary irradiation' OR 'external beam radiotherapy' OR 'external beam radiation therapy' OR EBRT)

AND

('overall treatment time' OR 'overall treatment times' OR 'overall treatment duration' OR 'total treatment time' OR 'total treatment duration' OR 'treatment time' OR 'treatment duration' OR 'prolonged overall treatment time' OR 'prolonged treatment time' OR 'prolonged treatment duration' OR 'protracted treatment' OR 'treatment prolongation' OR 'treatment protraction' OR 'treatment interruption' OR 'treatment interruptions' OR 'radiotherapy interruption' OR 'radiotherapy interruptions' OR 'radiation interruption' OR 'radiation interruptions' OR 'treatment break' OR 'treatment breaks' OR 'radiotherapy break' OR 'radiotherapy breaks' OR 'treatment gap' OR 'treatment gaps')

AND

('oncologic outcome' OR 'oncologic outcomes' OR 'oncological outcome' OR 'oncological outcomes' OR prognos* OR survival OR 'overall survival' OR 'cancer-specific survival' OR 'disease-specific survival' OR 'disease-free survival' OR 'progression-free survival' OR 'recurrence-free survival' OR recurren* OR 'local control' OR 'pelvic control' OR 'locoregional control' OR 'tumor control' OR 'tumour control' OR 'local failure' OR 'pelvic failure' OR 'locoregional failure' OR 'distant failure')

**3. Web of Science Core Collection**

**Database:** Web of Science Core Collection

**Date range:** from database inception to 1 August 2026

**Search field:** Topic (TS)

**Search strategy:**

TS=(("cervical cancer" OR "cervical carcinoma" OR "cancer of the cervix" OR "carcinoma of the cervix" OR "uterine cervical cancer" OR "uterine cervical carcinoma")

AND

(radiotherap* OR "radiation therapy" OR chemoradiotherap* OR chemoradiation OR radiochemotherap* OR "concurrent chemoradiotherapy" OR "concurrent chemoradiation" OR CCRT OR brachytherap* OR "intracavitary radiation" OR "intracavitary radiotherapy" OR "intracavitary irradiation" OR "external beam radiotherapy" OR "external beam radiation therapy" OR EBRT)

AND

("overall treatment time" OR "overall treatment times" OR "overall treatment duration" OR "total treatment time" OR "total treatment duration" OR "treatment time" OR "treatment duration" OR "prolonged overall treatment time" OR "prolonged treatment time" OR "prolonged treatment duration" OR "protracted treatment" OR "treatment prolongation" OR "treatment protraction" OR "treatment interruption" OR "treatment interruptions" OR "radiotherapy interruption" OR "radiotherapy interruptions" OR "radiation interruption" OR "radiation interruptions" OR "treatment break" OR "treatment breaks" OR "radiotherapy break" OR "radiotherapy breaks" OR "treatment gap" OR "treatment gaps")

AND

("oncologic outcome" OR "oncologic outcomes" OR "oncological outcome" OR "oncological outcomes" OR prognos* OR survival OR "overall survival" OR "cancer-specific survival" OR "disease-specific survival" OR "disease-free survival" OR "progression-free survival" OR "recurrence-free survival" OR recurren* OR "local control" OR "pelvic control" OR "locoregional control" OR "tumor control" OR "tumour control" OR "local failure" OR "pelvic failure" OR "locoregional failure" OR "distant failure"))

**4. Scopus**

**Database:** Scopus

**Date range:** from database inception to 1 August 2026

**Search field:** TITLE-ABS-KEY

**Search strategy:**

TITLE-ABS-KEY

(("cervical cancer" OR "cervical carcinoma" OR "cancer of the cervix" OR "carcinoma of the cervix" OR "uterine cervical cancer" OR "uterine cervical carcinoma")

AND

(radiotherap* OR "radiation therapy" OR chemoradiotherap* OR chemoradiation OR radiochemotherap* OR "concurrent chemoradiotherapy" OR "concurrent chemoradiation" OR CCRT OR brachytherap* OR "intracavitary radiation" OR "intracavitary radiotherapy" OR "intracavitary irradiation" OR "external beam radiotherapy" OR "external beam radiation therapy" OR EBRT)

AND

("overall treatment time" OR "overall treatment times" OR "overall treatment duration" OR "total treatment time" OR "total treatment duration" OR "treatment time" OR "treatment duration" OR "prolonged overall treatment time" OR "prolonged treatment time" OR "prolonged treatment duration" OR "protracted treatment" OR "treatment prolongation" OR "treatment protraction" OR "treatment interruption" OR "treatment interruptions" OR "radiotherapy interruption" OR "radiotherapy interruptions" OR "radiation interruption" OR "radiation interruptions" OR "treatment break" OR "treatment breaks" OR "radiotherapy break" OR "radiotherapy breaks" OR "treatment gap" OR "treatment gaps")

AND

("oncologic outcome" OR "oncologic outcomes" OR "oncological outcome" OR "oncological outcomes" OR prognos* OR survival OR "overall survival" OR "cancer-specific survival" OR "disease-specific survival" OR "disease-free survival" OR "progression-free survival" OR "recurrence-free survival" OR recurren* OR "local control" OR "pelvic control" OR "locoregional control" OR "tumor control" OR "tumour control" OR "local failure" OR "pelvic failure" OR "locoregional failure" OR "distant failure"))

**5. Cochrane Library**

**Database:** Cochrane Library

**Date range:** from database inception to 1 August 2026

**Search field:** Title/Abstract/Keyword

**Search strategy:**

("cervical cancer" OR "cervical carcinoma" OR "cancer of the cervix" OR "carcinoma of the cervix" OR "uterine cervical cancer" OR "uterine cervical carcinoma")

AND

(radiotherap* OR "radiation therapy" OR chemoradiotherap* OR chemoradiation OR radiochemotherap* OR "concurrent chemoradiotherapy" OR "concurrent chemoradiation" OR CCRT OR brachytherap* OR "intracavitary radiation" OR "intracavitary radiotherapy" OR "intracavitary irradiation" OR "external beam radiotherapy" OR "external beam radiation therapy" OR EBRT)

AND

("overall treatment time" OR "overall treatment times" OR "overall treatment duration" OR "total treatment time" OR "total treatment duration" OR "treatment time" OR "treatment duration" OR "prolonged overall treatment time" OR "prolonged treatment time" OR "prolonged treatment duration" OR "protracted treatment" OR "treatment prolongation" OR "treatment protraction" OR "treatment interruption" OR "treatment interruptions" OR "radiotherapy interruption" OR "radiotherapy interruptions" OR "radiation interruption" OR "radiation interruptions" OR "treatment break" OR "treatment breaks" OR "radiotherapy break" OR "radiotherapy breaks" OR "treatment gap" OR "treatment gaps")

AND

("oncologic outcome" OR "oncologic outcomes" OR "oncological outcome" OR "oncological outcomes" OR prognos* OR survival OR "overall survival" OR "cancer-specific survival" OR "disease-specific survival" OR "disease-free survival" OR "progression-free survival" OR "recurrence-free survival" OR recurren* OR "local control" OR "pelvic control" OR "locoregional control" OR "tumor control" OR "tumour control" OR "local failure" OR "pelvic failure" OR "locoregional failure" OR "distant failure")
